# Under-five Protein Energy Malnutrition Admitted at the University of In Nigeria Teaching Hospital, Enugu: a 10 year retrospective review

**DOI:** 10.1186/1475-2891-11-43

**Published:** 2012-06-14

**Authors:** Agozie C Ubesie, Ngozi S Ibeziako, Chika I Ndiokwelu, Chinyeaka M Uzoka, Chinelo A Nwafor

**Affiliations:** 1 Department of Paediatrics, Faculty of Medical Sciences, College of Medicine, University of Nigeria, Enugu, Nigeria; 2 Department of Paediatrics, University of Nigeria Teaching Hospital, Ituku/Ozalla, Enugu, Nigeria; 3 Department of Nutrition and Dietetics, University of Nigeria Teaching Hospital, Ituku/Ozalla, Enugu, Nigeria

**Keywords:** PEM, Under-five children, Case fatality, Co-morbidities, Admission, Enugu

## Abstract

**Objective:**

To determine the prevalence, risk factors, co-morbidities and case fatality rates of Protein Energy Malnutrition (PEM) admissions at the paediatric ward of the University of Nigeria Teaching Hospital Enugu, South-east Nigeria over a 10 year period.

**Design:**

A retrospective study using case Notes, admission and mortality registers retrieved from the Hospital’s Medical Records Department.

**Subjects:**

All children aged 0 to 59 months admitted into the hospital on account of PEM between 1996 and 2005.

**Results:**

A total of 212 children with PEM were admitted during the period under review comprising of 127 (59.9%) males and 85(40.1%) females. The most common age groups with PEM were 6 to 12 months (55.7%) and 13 to 24 months (36.8%). Marasmus (34.9%) was the most common form of PEM noted in this review. Diarrhea and malaria were the most common associated co-morbidities. Majority (64.9%) of the patients were from the lower socio-economic class. The overall case fatality rate was 40.1% which was slightly higher among males (50.9%). Mortality in those with marasmic-kwashiokor and in the unclassified group was 53.3% and 54.5% respectively.

**Conclusion:**

Most of the admissions and case fatality were noted in those aged 6 to 24 months which coincides with the weaning period. Marasmic-kwashiokor is associated with higher case fatality rate than other forms of PEM. We suggest strengthening of the infant feeding practices by promoting exclusive breastfeeding for the first six months of life, followed by appropriate weaning with continued breast feeding. Under-five children should be screened for PEM at the community level for early diagnosis and prompt management as a way of reducing the high mortality associated with admitted severe cases.

## Background

Globally, PEM continues to be a major health burden in developing countries and the most important risk factor for illnesses and death especially among young children [1]. The World Health Organization estimates that about 60% of all deaths, occurring among children aged less than five years in developing countries, could be attributed to malnutrition [2]. The improvement of nutrition therefore, is the main prerequisite for the reduction of high infant and under five mortality rates, the assurance of physical growth, social and mental development of children as well as academic achievement [3]. Sub-saharan Africa bears the brunt of PEM in the world. On the average, the PEM associated mortality in sub-Saharan Africa is between 25 and 35% [4,5]. In Nigeria, 22 to 40% of under-five mortality has been attributed to PEM [6]. PEM is also associated with a number of co-morbidities such as lower respiratory tract infections including tuberculosis, diarrhea diseases, malaria and anaemia [7,8]. These co-morbidities may prolong the duration of hospital stay and death among affected children.

There is a knowledge gap on the incidence and outcome of PEM seen in the Nigerian tertiary health facilities. In this study, the type of PEM among admitted under-five children, the associated morbidities, and duration of hospitalization and outcome at the University of Nigeria Teaching Hospital Enugu over a 10 year period is reviewed.

## Methods

### Setting

This study was conducted at the University of Nigeria Teaching Hospital, Enugu. Enugu is the capital of Enugu State, and is located in south-east Nigeria. It has an estimated population of about 3.3 million inhabitants according to the 2006 national census figures [9]. The main social and economic activities include small and medium scale trading, artisan works while a significant number of people are also employed in the various government institutions. The inhabitants are mainly Igbos and cut across all social-economic class. The main health-related problems seen among children in Enugu are diarrhea diseases, malaria and respiratory tract infections. The old site of the Teaching Hospital (where the hospital was cited during the period under review) had 700 bed capacity and provided specialty care in paediatrics, internal medicine, surgery and obstetrics & gynaecology as well as subspecialty care in over 50 other areas. The paediatric wards have 80 bed capacities. On the average 640 children (aged 0–18 years) were admitted into the paediatric wards annually and malnutrition accounted for nearly 3% of those admissions.

### Study design

This was a 10 year (1996–2005) retrospective quantitative study. The source documents for retrieving information were the admission and mortality registers of the hospital during the period under review. Available case notes/folders were also retrieved. A proforma was used to obtain relevant information. These information included date of admission and discharge, bio-data, clinical features, history of breast feeding, socio-economic status of the Caregiver, classification of malnutrition using Modified Wellcome Classifications, co-morbidities noted and, eventual outcome. Outcome was discharged from hospital, died while still in the hospital or discharged against medical advice. The advantage of using retrospective quantitative study was that reasonable sample size could be achieved in a relatively short time.

### Participants

A total of 7703 children were admitted during the period under review. All children aged six to 59 months admitted into the paediatric wards of UNTH Enugu between 1996 and 2005 with diagnosis of any form of PEM were included in this review. Their case files and/or documentations on hospital registers were retrieved from the Medical Records Unit of the hospital. Children with diagnosis of PEM but had in addition, other chronic conditions such as congenital heart diseases and cerebral palsy were excluded.

The outcome variables were recovery and discharged; death and discharge against medical advice. Recovery was defined as children, whose appetite has returned, gaining weight with resolution of clinical features. Death was defined based on hospital records and exclude those that may have died at home. Discharged against medical advice were those that did not meet the discharge criteria but whose Caregivers insisted on going home.

## Materials

The proforma for the study contained information on the age of the participants in months, sex, year of admission, diagnosis, co-morbidities, mode of breast feeding and duration, socio-economic status and outcome (recovered and discharged, discharged against medical advice or died). Socio-economic status of each child was determined using Oyedeji [10] classification that considers the highest educational attainment and occupation of the parents. The scoring is from I to V; social classes I and II were regarded as upper class, III as middle while IV and V constituted lower social class.

### Procedures

Relevant information was extracted from each retrieved case file and/or hospital registers and transferred into the proforma. Diagnosis of PEM was based on the Modified Wellcome Classification because it was the method used for clinical diagnosis by the clinicians. This classified PEM into kwashiorkor, underweight kwashiorkor, underweight, marasmus, marasmic kwashiorkor and there was also provision for unclassified PEM. Marasmus and the various forms of kwashiorkor are part of the recently defined Severe Acute Malnutrition (SAM) by the World Health Organization (WHO). The WHO defined SAM by a very low weight for height (below -3z scores of the median WHO growth standards), visible severe wasting or the presence of nutritional oedema [11,12]. Modified Wellcome classification uses weight for age and the presence or absence of oedema to classify PEM. The weights were measured using infant weighing scales (Waymaster) and stadiometers (Health Scale) depending on the age of the child. A total of 212 proforma were completed covering the entire period of the study.

### Diagnostic methods and clinical definition of co-morbidities

Diagnosis of HIV was made using Enzyme Linked Immunosorbent Assay [ELISA] and Westerblot. In children aged less than 18 months, positive antibody test was combined with clinical features to make presumptive diagnosis of HIV infection. Diagnosis of malaria was confirmed using blood film and bronchopneumonia using chest X-ray. Diarrhea was defined as passage of watery or loose stools or an increase in frequency above normal for a child. Severe anaemia was defined using a packed cell volume of less than 15%. Sepsis was defined as clinical features of systemic inflammatory response (fever, tachycardia, tachypnea, leukocytosis or leukopenia) associated with infection. Diagnosis of tuberculosis was made in the presence of chronic cough that have lasted for more three weeks supported by varied combination of the following: positive family history of tuberculosis, positive mantoux, suggestive chest X-ray and elevated erythrocyte sedimentation rate. Diagnosis of scabies was clinical based on the typical itching papular rash located at the intertrigous areas. Chronic suppurative otitis media and rickets were suspected clinically and confirmed by culture of ear swab and X-ray of the limbs respectively.

### Data analysis

The data were analyzed using SPSS version 19. Chi-square was used to test significant association of categorical variables: sex, age group, mortality rate and number of complications while One-way Analyis of Variables (ANOVA) was used to test for significant association of the continuous variable (mean hospitalization duration and mean number of complications). A p-value of less than 0.05 was regarded as significant and 95% confidence interval reported where indicated.

### Ethical approval

Ethical approval was obtained from the Health Research and Ethics Committee of the University of Nigeria Teaching Hospital, Enugu, Enugu State, Nigeria.

## Results

### Subjects

A total of 7703 children were admitted into the paediatric wards and 212 of them were cases of PEM during the period under review. This represented about 2.8% of the total paediatric admissions. One hundred and twenty seven (59.9%) were males while 85 (40.1) were females giving a male: female ratio of 1: 0.7. The age group studied was 6 to 59 months (under-5). The mean age of the participants was 15.4 ± 9.3 months.

### PEM and demography

**Figure 1:**
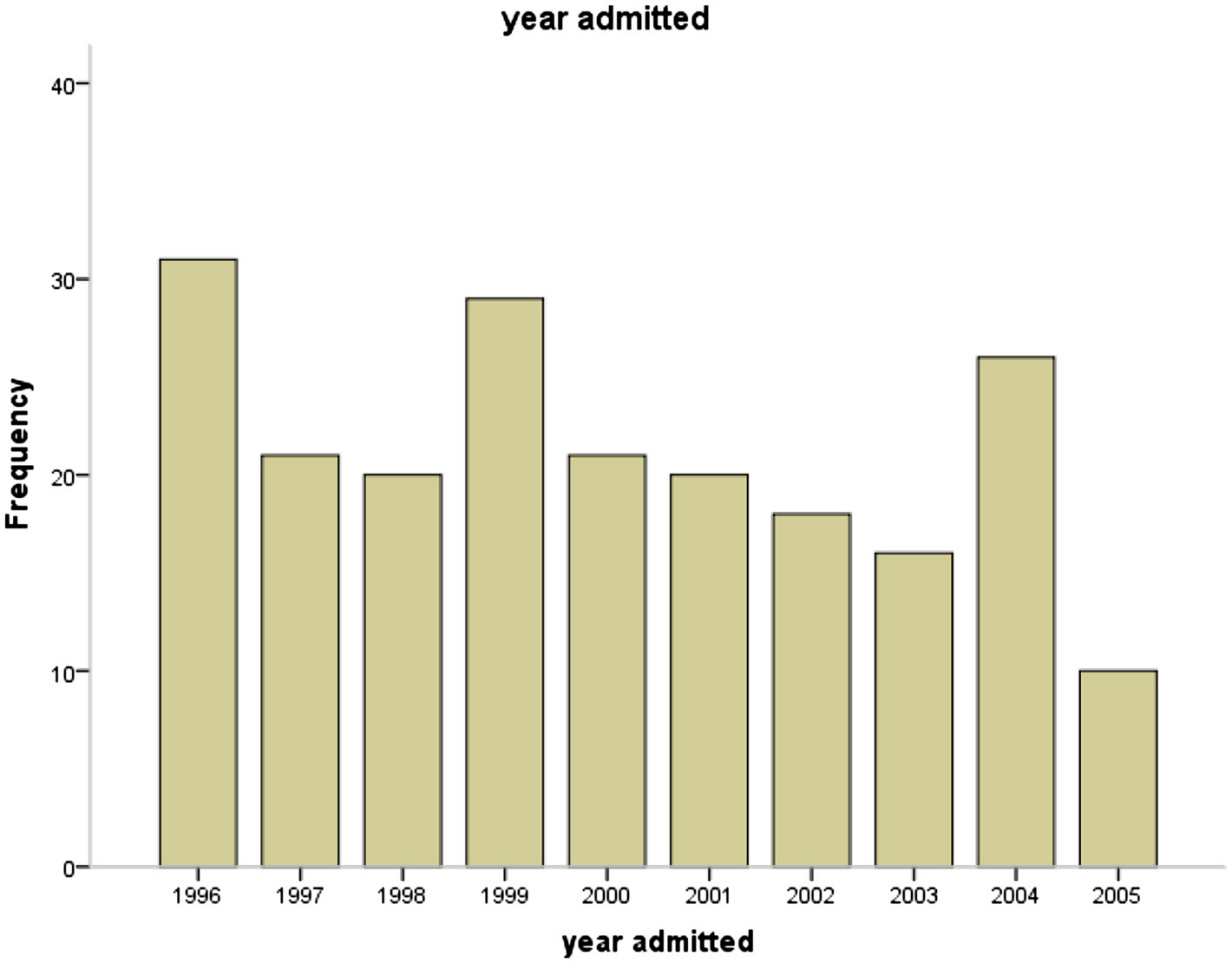
**Number of children admitted with PEM by year.** This figures describes the number of children admitted with PEM per year for the years reviewed.

PEM was most common among the age groups 6 to 12 and 13 to 24 months, and these accounted for 55.7% and 36.8% of the study population respectively. There was however, no statistically significant difference between the age groups and various forms of PEM as shown in Table 1(χ² = 19.38, df =16, p = 0. 249). The most common form of PEM noted in this review was marasmus (34.9%). Except for marasmic-kwashiokor, more males than females had more of all the various types although this was not statistically significant (χ² ^=^ 8.382, df =4, p = 0. 079) as shown in Table 2. Admissions for PEM were recorded more in 1996, 1999 and 2004 (15.1, 13.7 and 12.3% respectively), but there were no consistent pattern in the yearly admissions of children with PEM during the period under review (Figure 1).

**Table 1:** PEM admissions according to the age groups (months)

**PEM type**	**0-12 m (%)**	**13-24 m (%)**	**25-36 m (%)**	**37-48 m (%)**	**49-60 m (%)**
Kwashiokor	16 (13.6)	19 (24.4)	3 (33.3)	1 (33.3)	1 (25)
Underweight	11 (9.3)	6 (7.7)	0 (0)	0 (0)	0 (0)
Marasmic-kwash	6 (5.1)	8 (10.3)	0 (0)	1 (33.3)	0 (0)
Marasmus	48 (40.7)	24 (30.8)	2 (22.2)	0 (0)	0 (0)
Unclassified	37 (31.4)	21 (26.9)	4 (44.4)	1 (33.3)	3 (75)
**Total**	**118 (100)**	**78 (100)**	**9 (100)**	**3(100)**	**4 (100)**

χ² = 19.38, df =16, *P* = 0. 249.

**Table 2:** Sex Distribution of the Clients

**PEM type**	**Male (%)**	**Female (%)**
Kwashiokor	30 (23.6)	10 (11.8)
Underweight	12 (9.4)	5 (5.9)
Marasmic-kwash	6 (4.7)	9 (10.6)
Marasmus	44 (34.6)	30 (35.3)
Unclassified	35 (27.6)	31 (36.5)
**Total**	**127 (100)**	**85 (100)**

χ² = 8.382, df =4, *P* = 0. 07.

### Confounding variables for PEM

Record for associated co-morbidities was available in only 66 subjects. Diarrhea and malaria were the most common associated conditions in 72.7% and 43.9% of the children respectively. Other notable associated conditions were sepsis (37.9%), severe anaemia (24.2%), bronchopneumonia (16.7%) and tuberculosis (12.1%) as shown in Table 3. Information on the educational level and occupation of the parents was available in only 33 of the reviewed cases. Among them, 69.4% belonged to the lower social class, 19.4% to middle class while 5.6% was of the upper class. The predominant infant feeding in the first six months was breast milk and water for 0 to 3 months (48.6%), breast milk and water for 4 to 6 months (24. 3%), exclusive breast feeding for up to 3 months (18.9%) and use of breast milk substitutes (8.1%) as shown in Table 4. These feeding methods were followed by weaning with pap gruel that was variably fortified for the children.

**Table 3:** The associated co-morbidities seen among patients

**Co-morbidity**	**Frequency**
	**(%)**
Diarrhea	48 (72.2)
Malaria	29 (43.9)
Sepsis	25 (37.9)
Severe anaemia	16 (24.2)
Bronchopneumonia.	11 (16.7)
HIV	9 (13.6)
Tuberculosis	8 (12.1)
Scabies	2 (3.0)
Chronic suppurative otitis media	1 (1.5)
Rickets	1 (1.5)
Keratomalacia	1 (1.5)

The table shows the associated co-morbidities noted in the patients.

**Table 4:** Prevalence of PEM by breastfeeding pattern

**Breastfeeding pattern**	**Prevalence**	**95% Confidence Intervals**
	**(%)**	
Exclusive breast feeding for 0–3 months	18.9	11.2 - 26.6
Predominant breastfeeding 0–3 months	48.6	38.8 – 58.4
Predominant breastfeeding 4–6 months	24.3	15.9 – 32.7
Breast milk substitutes	8.1	2.7 – 13.5

The table shows the prevalence of the various pattern of feeding for the children during their early infancy. The 95% confidence interval is also reported.

### Prognostic indicators

The duration of hospitalization was available in only 84 subjects and ranged from 0 to 62 days. The mean duration of hospitalization was 16 ± 15 days. Kwashiokor patients had the highest mean hospitalization days of 19.15 days while marasmic and underweight patients had the least days of 14.52 and 14.55 days respectively. There was no statistically significant difference in the mean hospitalization days for the various types of PEM (F = 0.317, df =4, *P* = 0. 866). A total of 85 (40.1%) children died while on admission, 124 (58.5%) recovered and were discharged home while 3 (1.4%) were discharged against medical advice. Mortality was higher among the males (50.9%) than females (34.1%) although this was not statistically significant (χ² = 0.723, df =2, *P* = 0. 697). Most of the deaths were recorded in the age groups 0–12 (55.3%) and 13–24 (36.5%) months although this difference was not statistically significant (χ² = 10.98, df =8, p = 0. 203). The marasmic-kwashiokor and unclassified groups had higher mortality rates (53.3% and 54.5% respectively) than the marasmus (37.8%) or kwashiorkor groups (30%). There was a statistically significant difference in the mortality rates of the various types of PEM as shown in Table 5 (χ² = 17.26, df =4, p = 0. 002) The number of complications ranged from none to four. Kwashiokor has the highest mean number of complications (2.06) while unclassified had the least number of 1.26. There was a statistically significant difference in the number of complications and the various PEM (F = 8.92, df =4, *P* <0.05)

**Table 5:** Prognostic indicators of protein-energy malnutrition

**Prognostic indicator**	**Kwash (%)**	**UWM (%)**	**MK (%)**	**Marasmus (%)**	**Unclassified (%)**	***P-*value**
Mean no of complications	2.06	2.00	1.83	1.67	1.26	*P* < 0.05
Mean hospitalization days	19.15	14.55	16.2	14.52	16.33	0.866
Mortality rate	12 (30)	1 (5.9)	8 (53. 3)	27 (36.5)	36 (54.5)	0.002

*Kwash* = Kwashiokor; *UWM* = Underweight malnutrition, *MK* = Marasmic kwashiokor. The table shows some prognostic indicators: mean number of complications, mean hospitalization days and mortality rates of the various types of PEM.

## Discussion

### Presenting features

There was no significant difference between the two genders from our review. This agrees with an earlier review in Maputo by Cartmell et al. [13]. The age group 6 to 24 months accounted for 92.5% of the total number of children admitted for PEM. In previous Nigerian and Zambian studies, an approximate 64% of admitted cases of PEM were less than two years [4,14] while Cartmell et al. [13] documented a mean age of 21.7 months in the Maputo study. The reasons for the high number of cases of PEM among the age group 6 to 24 months could be due to a number of factors including low rate of exclusive breast feeding as documented in our review as well as poor weaning and feeding practices. Marasmus was the most common type of PEM noted in our review (34.9%) beside the unclassified cases. Gernaat et al. [4] also found more of marasmic patients among males aged less than a year in their review. Kwashiorkor and marasmus were the most common types among admitted children in Maputo in 1983 but changed to Kwashiokor and marasmic-kwashiokor in 2001 according to the review by Cartmell et al. [13]. This may imply that there is no consistent geographical pattern that predicts the type of PEM a child will manifest but rather depends on interplay of factors.

### High PEM associated mortality

The overall mortality in our study was 40.1% which although lower than the WHO estimated 60% [2] is still very high. Studies conducted in various parts of Africa have documented unacceptable high mortality rates among children admitted for PEM. In Oshogbo, South West Nigeria, Ibekwe and Ashworth [6] documented an average mortality rate of 22% over a five year period among 803 children admitted for PEM in a Nutritional Rehabilitation Center. Similarly, in a hospital based study in north-eastern Zambia, involving children below the age of five years, Gernaat *et al.*[4] documented an overall mortality rate of 25.8% among 288 children admitted for various types of severe/complicated malnutrition . Higher mortality rate for marasmic kwashiorkor than marasmus or kwashiorkor was noted in this review. Gernaat *et al.*[4] noted similar finding in their review among Zambian children admitted and managed for PEM. This reason for this is unclear. However, Ibekwe and Ashworth [6] did note that PEM associated mortality among oedematous patients was significantly higher compared to those with marasmus. It can be argued therefore, that presence of oedema in a malnourished child connotes poor prognosis. The mean duration of hospitalization was 16 days which is similar to 13.1 and 14.3 days reported by Cartmell et al. [13] but differs from the 35 days reported by Ibekwe and Ashworth [6]. Both this review and the study by Cartmell *et al.* were hospital based while that of Ibekwe and Ashworth was conducted in a Nutrition Rehabilitation Center. The pressure on bed spaces in a hospital setting could have contributed to earlier discharges in hospital settings.

### Associated risk factors for PEM

Our review noted that PEM was more common among children from the lower social class (69.4%) and those predominantly breast fed for three months or less (48.6%) compared to exclusively breast fed children (18.9%). The reason for this may not be unconnected to the fact that poor families have low purchasing power for adequate nutritious foods for their families. Illiteracy on the other hand, may influence feeding practices. The low rate of exclusive breast feeding noted in this review despite the Baby Friendly Initiatives is also very worrisome. Poverty and illiteracy as risk factors for PEM have been documented in the literature. . In a case control study conducted in Dhaka, Bangladesh which involved children aged six to 24 months, Nahar *et al.*[15] compared 507 children with weight-for-age z-score (WAZ) < −3 matched for age, sex and place of residence with 500 children whose weight-for-age z-score (WAZ) were > −2.5 . They documented that severely-underweight children were more likely to have: undernourished poorly educated teenage mothers, history of shorter duration of predominant breastfeeding, and fathers who were poorly educated and unskilled day-labourers [15].

Diarrhea, malaria, sepsis and severe anaemia were the most prevalent associated co-morbidities from our review in that order. In Maputo, the most prevalent co-morbidities associated with PEM by Cartmell et al. were anaemia, bronchopneumonia, malaria and diarrhea. The prevalence of human immune deficiency virus (HIV) from our review was 13.6% and this compares to a prevalence of 12% in the Maputo study. This finding underscored the high rate of HIV infection among children with severe forms of PEM and the need to routinely screen such children for HIV when they present at a health facility.

## Conclusions

Younger children aged less than two years accounted for most of the admissions in this review. Marasmic-kwashiokor was associated with higher case fatality rate than other types of PEM. There is need therefore to strengthen the infant feeding practices by promoting exclusive breastfeeding for the first 6 months of life, followed by appropriate weaning with continued breast feeding till second year of life. PEM was associated with high rate of mortality in this hospital setting and preventive strategies need to be emphasized instead.

### Limitation and strength

This is a retrospective study and was not designed to optimize the risk factors and outcome of PEM but relied on data from previous documentations. At best, it may be a fair representation of some of the factors associated with PEM in a typical Nigerian teaching hospital. It is however the first paper to review PEM managed at a Nigeria tertiary health facility over a 10 year period.

## Competing interest

The authors declare that they have no competing interests.

## Authors’ contributions

ACU, CMU and CAN designed the study and collected the data including analysis. NSI, ACU and CCN did the literature review and drafted the initial manuscript. All the authors read and approved the final version of the manuscript.

## Authors’ information

ACU is a Lecturer in the Department of Paediatrics, College of Medicine, University of Nigeria and also an Honorary Consultant in the Department of Paediatrics, University of Nigeria Teaching Hospital, Ituku/Ozalla, Enugu. NSI is a Senior Lecturer in the Department of Paediatrics, College of Medicine, University of Nigeria and also an Honorary Consultant in the Department of Paediatrics, University of Nigeria Teaching Hospital Ituku/Ozalla, Enugu. CCU is the Head of the Department of Nutrition and Dietetics, University of Nigeria Teaching Hospital Ituku/Ozalla, Enugu.

## References

[B1] Muller O Krawinkel M Malnutrition and health in developing countries CMAJ 2005 173 3 279 286 Available from: http://www.cmaj.ca/cgi/content/full/173/3/279. (Accessed March 2, 2011) 10.1503/cmaj.050342 16076825 PMC1180662

[B2] Faruque ASG Ahmed AMS Ahmed T Islam MM Hossain MI Roy SK Alam N Kabir I Sack DA Nutrition: Basis for Healthy Children and Mothers in Bangladesh J Health Popul Nutr 2008 26 3 325 339 [Online]. Available from: http://www.ncbi.nlm.nih.gov.ezproxy.liv.ac.uk/pmc/articles/PMC2740711/?tool=pmcentrez. (Accessed July 8, 2011) 18831228 10.3329/jhpn.v26i3.1899 PMC2740711

[B3] Anwar F Khomsan A Sukandar D Riyadi H Mudjajanto ES High participation in posyandu nutrition program improved children nutritional status Nutr Res Pract 2010 4 3 208 214 [Online]. Available from: http://www.ncbi.nlm.nih.gov.ezproxy.liv.ac.uk/pmc/articles/PMC2895701/?tool=pmcentrez. (Accessed July 15, 2011) 10.4162/nrp.2010.4.3.208 20607066 PMC2895701

[B4] Gernaat HB Dechering WH Voorhoeve WH Mortality in Severe Protein-energy Malnutrition at Nchelenge, Zambia J Trop Pediatr 1998 44 4 211 217 [Online]. Available from: http://tropej.oxfordjournals.org.ezproxy.liv.ac.uk/content/44/4/211.abstract. (Accessed March 3, 2011) 10.1093/tropej/44.4.211 9718906

[B5] Rutherford GW Mahanjane AE Morbidity and Mortality in the Mozambican Famine of 1983: Prevalence of Malnutrition and Causes and Rates of Death and Illness among Dislocated Persons in Gaza and Inhambane Provinces J Trop Pediatr 1985 31 143 149 4020936 10.1093/tropej/31.3.143

[B6] Ibekwe VE Ashworth A Management of protein energy malnutrition in Nigeria: an evaluation of the regimen at the Kersey Nutrition Rehabilitation Center, Nigeria Trans R Soc Trop Med Hyg 1994 88 594 595 10.1016/0035-9203(94)90177-5 7992350

[B7] le Roux1 IM le Roux K Scott WC Greco EM Desmond KA Mbewu N Rotheram-Borus MJ Home visits by neighborhood Mentor Mothers provide timely recovery from childhood malnutrition in South Africa: results from a randomized controlled trial Nutr J 2010 9 56 [Online]. Available from: http://www.nutritionj.com/content/9/1/56. (Accessed January 6, 2012) 10.1186/1475-2891-9-56 21092178 PMC3002292

[B8] Ejaz MS Latif N Stunting and micronutrient deficiencies in malnourished children J Pak Med Assoc 2010 60 7 543 547 [Online]. Available from: http://www.jpma.org.pk/full_article_text.php?article_id=2165. (Accessed January 6, 2012) 20578603

[B9] Enugu population National population commission. Provisional census figures Official Gazette Federal Republic Nigeria 2007 24 B-185

[B10] Oyedeji GA Socioeconomic and cultural background of hospitalised children in Ilesha Niger J Paediatr 1985 12 11

[B11] Gernaat HB Voorhoeve WH A New Classification of Acute Protein Energy Malnutrition J Trop Pediatr 2000 46 96 106 [Online]. Available from: http://tropej.oxfordjournals.org.ezproxy.liv.ac.uk/content/46/2.toc. (Accessed July 15, 2011) 10.1093/tropej/46.2.97 10822936

[B12] WHO Severe Acute Malnutrition 2011 [Online]. Available from: http://www.who.int/nutrition/topics/malnutrition/en/index.html. (Accessed July 16, 2011)

[B13] Cartmell E Natalal H Francois I Ferreira MH Grahnquist L Nutritional and Clinical Status of Children Admitted to the Malnutrition Ward, Maputo Central Hospital: A Comparison of Data from 2001 and 1983 J Trop Pediatr 2005 51 2 102 105 10.1093/tropej/fmh088 15677369

[B14] Nnakwe N The effect and causes of protein-energy malnutrition in Nigerian children Nutr Res 1995 15 6 785 794 10.1016/0271-5317(95)00044-J

[B15] Nahar B Ahmed T Brown KH Hossain MI Risk Factors Associated with Severe Underweight among Young Children Reporting to a Diarrhoea Treatment Facility in Bangladesh J health Popul Nutr 2010 28 5 476 483 [Online]. Available from: http://www.ncbi.nlm.nih.gov.ezproxy.liv.ac.uk/pmc/articles/PMC2963770/?tool=pmcentrez. (Accessed July 9, 2011) 20941899 10.3329/jhpn.v28i5.6156 PMC2963770

